# Propofol Decreases Endoplasmic Reticulum Stress–Mediated Apoptosis in Retinal Pigment Epithelial Cells

**DOI:** 10.1371/journal.pone.0157590

**Published:** 2016-06-16

**Authors:** Xuezhi Zhou, Yantao Wei, Suo Qiu, Yue Xu, Ting Zhang, Shaochong Zhang

**Affiliations:** State Key Laboratory of Ophthalmology, Zhongshan Ophthalmic Center, Sun Yat-sen University, Guangzhou, Guangdong, China; University of Hong Kong, HONG KONG

## Abstract

Age-related macular degeneration (AMD) is the major cause of loss of sight globally. There is currently no effective treatment available. Retinal pigment epithelial (RPE) cells are an important part of the outer blood-retina barrier and their death is a determinant of AMD. Propofol, a common clinically used intravenous anesthetic agent, has been shown to act as an efficacious neuroprotective agent with antioxidative and anti-inflammatory properties *in vivo* and *in vitro*. However, little is known about its effects on RPE cells. The purpose of our research was to investigate whether propofol could protect RPE cells from apoptosis through endoplasmic reticulum (ER) stress–dependent pathways. To this end, prior to stimulation with thapsigargin (TG), ARPE-19 cells were pretreated with varying concentrations of propofol. A protective effect of propofol in TG-treated ARPE-9 was apparent, TUNEL and flow cytometric assays showed decreased apoptosis. We further demonstrated that propofol pretreatment attenuated or inhibited the effects caused by TG, such as upregulation of Bax, BiP, C/EBP homologous protein (CHOP), active caspase 12, and cleaved caspase 3, and downregulation of Bcl2. It also decreased the TG-induced levels of ER stress–related molecules such as p-PERK, p-eIF2α, and ATF4. Furthermore, it downregulated the expression of nuclear factor κB (NF-κB). This study elucidated novel propofol-induced cellular mechanisms for antiapoptotic activities in RPE cells undergoing ER stress and demonstrated the potential value of using propofol in the treatment of AMD.

## Introduction

Age-related macular degeneration (AMD) is the leading cause of blindness and irreversible sight loss among the elderly in industrialized nations. Apoptosis and progressive degeneration of the retinal pigment epithelium (RPE) is an early event related to AMD [1, 2]. The RPE is a monolayer of pigmented cells attached to the overlying retinal ocular cells and underlying choroid, a layer that nourishes the retina [3]. Hypoxic or ischemic conditions can easily affect RPE cells that are located near the choroidal capillaries [4]. It is accepted that apoptosis of RPE cells is an important aspect of AMD pathogenesis. Although a diverse range of pro-apoptotic factors may be involved, oxidative stress may play a more important role.

ER stress and unfolded protein response (UPR) is induced by oxidative stress in RPE cells [5]. Recently, endoplasmic reticulum (ER) stress has been recognized as another key risk factor that exacerbates pathogenic progression of AMD [6]. UPR can be induced by an accumulation of unfolded proteins in the ER lumen and is governed by three ER membrane-bound sensors, PERK, ATF6, and IRE1α, which influence three distinct signaling pathways[7]. The ER-protective function of UPR is coordinated through transcriptional activation of chaperone genes, activation of the ER-associated degradation machinery, and inhibition of protein synthesis [8]. If the protective role of UPR is not achieved, apoptosis is induced to eliminate impaired RPE cells.

Propofol (2, 6-disopropylphenol) is an intravenous short-acting anesthetic small-molecule agent widely applied to induce and maintain anesthesia [9]. Propofol also exerts antiemetic, immune-modulatory, anxiolytic, and analgesic effects [10, 11]. Significantly, propofol acts as an efficacious neuroprotective agent in different models both *in vivo* and *in vitro* [12–16]. It has demonstrated antioxidant properties through the reduction of N-methyl-D-aspartate-induced increase in superoxide anion levels in cultured rat forebrain neurons [17] and shown neuroprotective effects in astrocytes by increasing HO-1 expression and attenuating SIN-1-mediated DNA ladderization and caspase 3 activation [18]. Studies have also shown that propofol can suppress lipopolysaccharide-induced production of inflammatory substances, including interleukin (IL)-1 beta, IL-10, thromboxane B(2), prostaglandin E(2), cyclooxygenase (COX) enzyme, and tumor necrosis factor-alpha (TNF-alpha), in microglial cells [19]. In previous research, propofol has been considered as a neuroprotective drug against apoptosis, inflammation, and oxidative stress in central nervous system diseases[20–22]. Considering that RPE cells are of neuronal origin, it is reasonable to determine whether propofol could also have a protective effect on RPE cells.

Therefore, in this study, we investigated whether propofol had an anti-apoptotic and protective function on ARPE-19 cells treated by thapsigargin (TG). We also explored whether ER stress induced by TG could be attenuated by propofol by modulating the PERK/eIF2α pathway in ARPE-19 cells.

## Materials and Methods

### Cell culture

The human retinal pigment epithelia cell line ARPE-19 was obtained from the American Type Culture Collection (Zhongyuan Company, Beijing, China). For experiments, the cells were seeded at 100,000/cm^2^ and grown in Dulbecco’s Modified Eagle’s Medium/Hams F12 (Grand Island, NY, USA) with 10% fetal bovine serum (Grand Island, NY, USA) and 100 μg/ml streptomycin (Beyotime, Haimen, China). Cultures were maintained at 37°C in a humidified atmosphere of 95% air and 5% CO_2_. Trypsinization (Grand Island, NY, USA) was used to passage the ARPE-19 cells every 3 days. Propofol (Sigma-Aldrich, MO, USA) was dissolved in DMSO shortly before use and an equal volume of DMSO was used as control. A range of concentrations of propofol was added to the cells for 12 h, prior to the addition of TG (1 μM) for 24 h (Sigma-Aldrich, MO, USA).

### MTT assay

ARPE-19 cells were plated in 100 μl of cell suspension (1–10×10^3^ cells/well) in 96-well plates. Experimental treatments were carried out after 24 h. (3-(4,5-Dimethylthiazol-2-yl)-2,5-diphenyltetrazolium bromide (MTT; Sigma-Aldrich, MO, USA) solution was added to each well after treatment, followed by incubation of an additional 4 h. The crystals were solubilized using 150 μl of dimethyl sulfoxide (DMSO) after replacement of the MTT solution. A microplate reader was used to measure the optical density value of each well at 490 nm. Five replicates were used for each sample, and the experiment was repeated three times.

### TUNEL analysis

For the terminal deoxynucleotidyl transferase (TdT)-mediated dUTP nick end labeling (TUNEL) assay (Biotool), ARPE-19 cells were fixed with freshly prepared 4% paraformaldehyde solution in PBS for 25 min at 4°C, washed with fresh PBS twice for 5 min, and permeabilized by immersing cell slides in 0.2% TritonX-100 solution in PBS for 5 min at room temperature. Thereafter, cells were washed in PBS for 5 min, incubated with 100 μl of 1× equilibration buffer at room temperature for 5–10 min. The cells were then incubated with 50 μl of the reaction mixture at 37°C for 60 min and washed 3 times with PBS. The cell nuclei were stained with 4',6-diamidino-2-phenylindole (DAPI) for 15 min, and washed in PBS for 5 min at room temperature. Finally, the cells were mounted onto coverslips. Cell images were captured with a ZEISS LSM 510 confocal microscope at 488 nm.

### Flow cytometric analysis of annexin V-fluorescein isothiocyanate

An annexin V-fluorescein isothiocyanate (FITC) apoptosis detection kit (Becton-Dickinson, CA, USA) was used to assess apoptosis. After experimental treatments, ARPE-19 cells were collected and suspended in 1× binding buffer containing annexin V-FITC and propidium iodide (PI) according to the manufacturer instructions. Fluorescence was measured with a FACS scan flow cytometry (Becton-Dickinson, San Jose, CA, USA).

### Western blot analysis

ARPE-19 cells were harvested at indicated time-points, washed with PBS, and lysed using RIPA lysis buffer (Beyotime, Haimen, China) followed by SDS-PAGE. Proteins were transferred to PVDF membranes (Millipore, Bedford, MA) and the membranes were incubated for 1 h with 5% non-fat milk in TBST at room temperature. Then, the proteins were probed with anti-PERK antibody (1:200, sc-32577, Santa Cruz Biotechnology), anti-p-PERK antibody (1:200, sc-377400, Santa Cruz Biotechnology), anti-p-eIF2α antibody (1:1,000, 3597, Cell Signaling Technology), anti-ATF4 antibody (1:1,000, 11815, Cell Signaling Technology), anti-active caspase 12 antibody (1:1,000, ab62484, Abcam), anti-cleaved caspase 3 antibody (1:1,000, 9964, Cell Signaling Technology), anti-BiP antibody (1:1,000, 3177, Cell Signaling Technology), anti-ERK1/2 antibody (1:800, 9102, Cell Signaling Technology), anti-p-ERK1/2 antibody (1:800, 4370, Cell Signaling Technology), and anti-Bcl2 antibody (1:800, 15071, Cell Signaling Technology). Bands were visualized with HRP-conjugated goat anti-mouse or goat anti-rabbit secondary antibody and the ECL Western Blotting Detection System.

### Confocal laser microscopy

ARPE-19 cells grown on glass coverslips were treated with propofol for different times and/or TG. After treatment, the cells were fixed with 4% paraformaldehyde for 15 min after washing in PBS for 3 times. Then, 1% BSA and 0.1% Triton X-100 in PBS were used to incubate the coverslips for 30 min. Then, the cells were treated with antibodies against p-eIF2α (1:400, 3597, Cell Signaling Technology), eIF2α (1:400, 5324, Cell Signaling Technology), ATF4 (1:400, 11815, Cell Signaling Technology), active caspase 12 (1:500, ab62484, Abcam), cleaved caspase 3 (1:400, 9964, Cell Signaling Technology), BiP (1:400, 3177, Cell Signaling Technology), CHOP (1:500, ab11419, Abcam), Bcl2 (1:400, 15071, Cell Signaling Technology), and Bax (1:500, ab77566, Abcam) for 2 h at room temperature. After the coverslips were washed in PBS, they were incubated with anti-rabbit or anti-mouse secondary antibody (1:400, 4412, Cell Signaling Technology). Cells nuclei were stained with DAPI (5 mg/ml) for 15 min. The coverslips were mounted with anti-fade mounting medium after washing in PBS. Images were obtained using a Zeiss Confocal Spectral Microscope (Carl Zeiss, Jena, Germany).

### Ca^2+^ assay

The cell-permeable calcium-sensitive fluorescent dye Fluo-3/AM (Beyotime, Haimen, China) was used to test the free cytosolic calcium levels. Cells were washed with PBS, and then were incubated with 5 μM Fluo-3/AM at 37°C for 1 h in the dark after the treatment with TG and propofol for 12 h. Then cells were washed with PBS for 3 times and fluorescence of Fluo-3 combined with cytosolic calcium was analyzed by FACS scan flow cytometry (Becton-Dickinson, San Jose, CA, USA).

### Statistical analysis

These experiments were performed at least 3 times. Statistical analysis was performed by Student’s t-test and ANOVA; *p*<0.05 was considered statistically significant.

## Results

### Effect of propofol on cytotoxicity in TG-induced ARPE-19 cells

**Fig 1:**
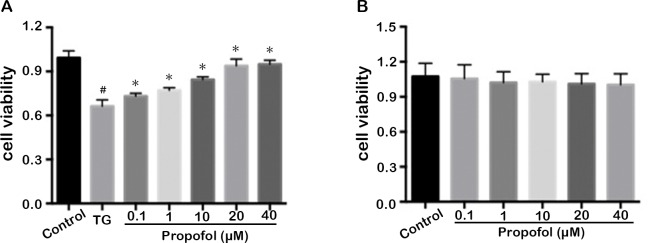
Propofol protect ARPE-19 cells against the damage caused by ER stress inducer TG. (A) Cell viability was measured in TG-treated ARPE-19 cells by pretreatment with various concentrations of propofol. ARPE-19 cells were pretreated with propofol for 24 h at the indicated concentration and follow with 1μM TG for 12 h. The Data are presented as the mean ± SEM from three independent experiments. Statistical analysis was performed by one-way analysis of variance with all pairwise multiple comparison procedures done by Tukey test. ^#^*p*<0.05 indicates TG group versus control group; *p<0.05 indicates propofol group versus TG group. (B) Cell viability was measured in ARPE-19 cells after treatment with various concentrations of propofol for 24 h. The data are presented as the mean ± SEM from three independent experiments. Statistical analysis was performed by one-way analysis of variance with all pairwise multiple comparison procedures done by Tukey test. ^#^*p*<0.05 indicates TG group versus control group; *p<0.05 indicates propofol group versus TG group.

Differences in cell viability, as detected in the MTT assay, were not apparent in ARPE-19 cells after incubation with various concentrations of propofol (0–40 μM) for 24 h (Fig 1B). This result indicated that propofol had no toxic effect on ARPE-19 cells. Propofol pretreatment (0–40 μM) for 24 h before TG exposure resulted in improved cell viability associated with an increasing concentration of propofol (Fig 1A). This result demonstrated that propofol could protect ARPE-19 cells from apoptosis caused by TG and that the protective potency was strongest at the 40 μM concentration.

### Effect of propofol on apoptosis of TG-treated ARPE-19 cells

**Fig 2:**
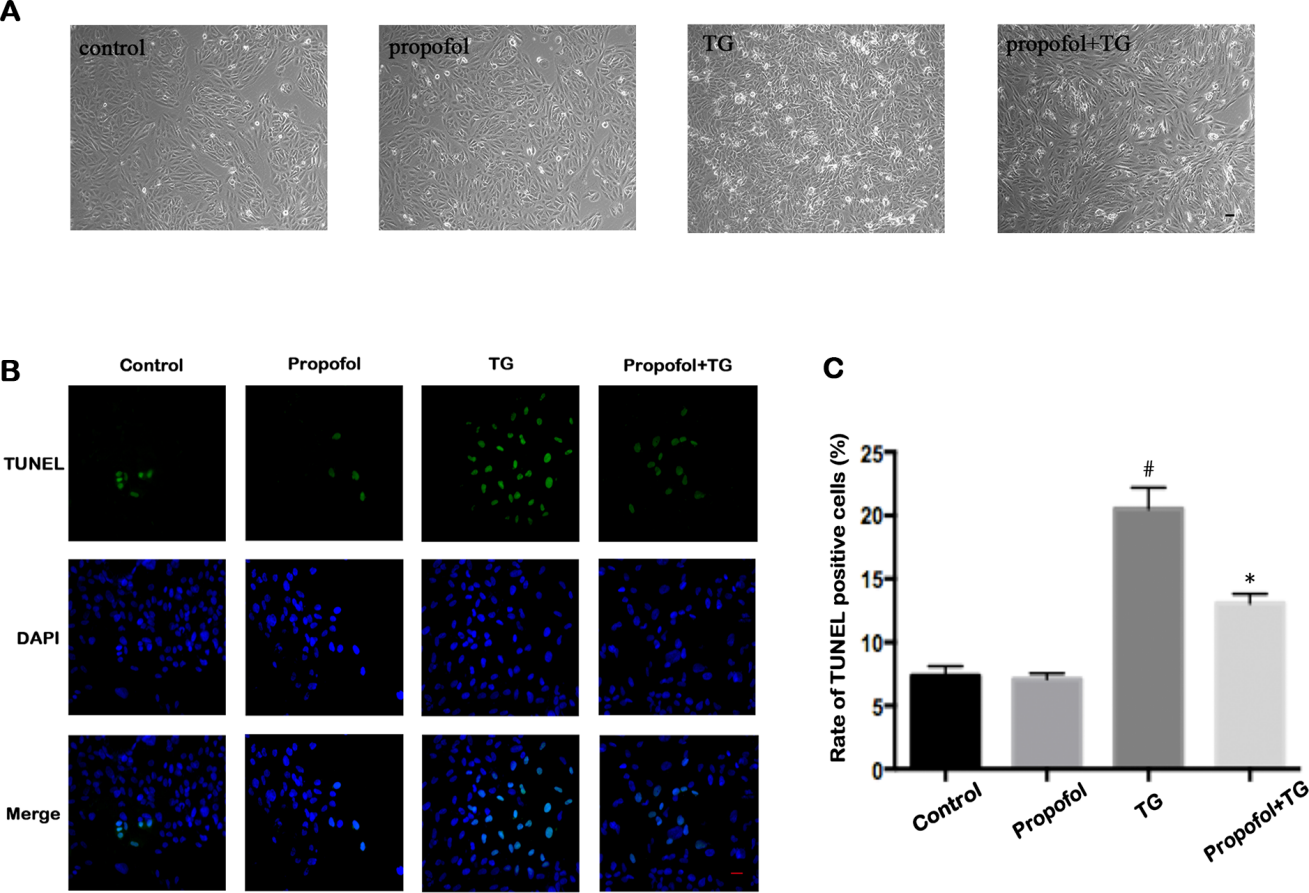
TG-induced ARPE-19 cells apoptosis was inhibited by propofol. (A) Representative phase-contrast images after propofol, TG, and propofol+TG treatment in ARPE-19 cells. Propofol group cells were treated with 40 μM of propofol for 24 h; TG group cells were treated with 1 μM of TG for 12 h; Propofol+TG group cells were treated with 40 μM propofol for 24 h and 1 μM TG for 12 h. Scale bar = 50 μm (B-C) Apoptosis of ARPE-19 cells in different groups was measured by TUNEL assay. Propofol reduced ARPE-19 cells apoptosis when compared with the TG group. ARPE-19 cells were precubated for 24 h with 40 μM propofol, then treated with 1 μM TG for 12 h. The data are presented as the mean ± SEM from three independent experiments. Statistical analysis was performed by one-way analysis of variance with all pairwise multiple comparison procedures done by Tukey test. ^#^*p*<0.05 versus control group. *p<0.05 versus TG group. Scale bar = 20 μm

**Fig 3:**
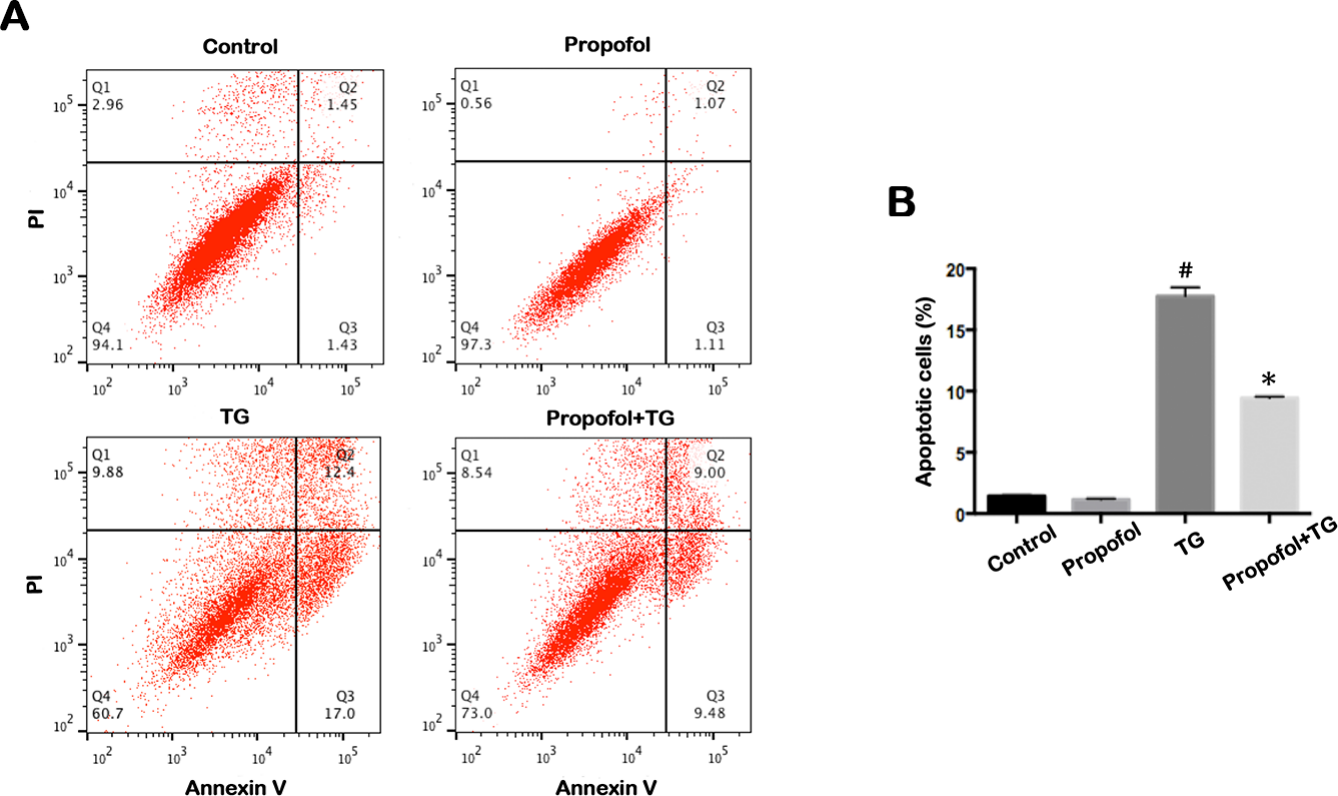
Propofol inhibits ARPE-19 cells apoptosis by Annexin V/PI staining. (A) Apoptotic cells in control group, Propofol group, TG group and Propofol+TG group were analyzed by Flow cytometry. The percentages of apoptosis cells were 1.43, 1.11, 17.0 and 9.48%. ARPE-19 cells were precubated for 24 h with 40 μM propofol, then treated with 1 μM TG for 12 h. (B) Apoptosis was higher in the TG group when compared with the control group. Apoptosis in the Propofol+TG group was lower than that in the TG group. The data are presented as the mean ± SEM from three independent experiments. Statistical analysis was performed by one-way analysis of variance with all pairwise multiple comparison procedures done by Tukey test. ^*#*^*p*<0.05 versus control group. *p<0.05 versus TG group.

To investigate the effect of propofol in protection of ARPE-19 cells, TG-treated ARPE-19 cells were pretreated with propofol. The morphologic images conformed the protective effect of propofol against TG in ARPE-19 cells (Fig 2A). As shown in the TUNEL analysis depicted in Fig 2B and 2C, TG-treated ARPE-19 cells exhibited a significantly greater proportion of apoptosis when compared with the control group. However, the rate of TUNEL-positive ARPE-19 cells was not significantly different between the control and propofol treatment groups. The frequency of cells undergoing apoptosis due to TG treatment significantly reduced among the cells pretreated with propofol (40 μM). Evaluation of the apoptosis rates, as measured by the flow cytometry method, was consistent with this finding (Fig 3A and 3B). TG caused a obvious increase in apoptosis (17.0 versus 1.43%) compared to control group. Treating with propofol before TG significantly decreased apoptosis after TG (9.48 versus 17.0%). Moreover, no apparent differences were found among control and propofol group in normal conditions (1.11 versus 1.43%).

### Effect of propofol on apoptosis-associated proteins in TG-treated ARPE-19 cells

**Fig 4:**
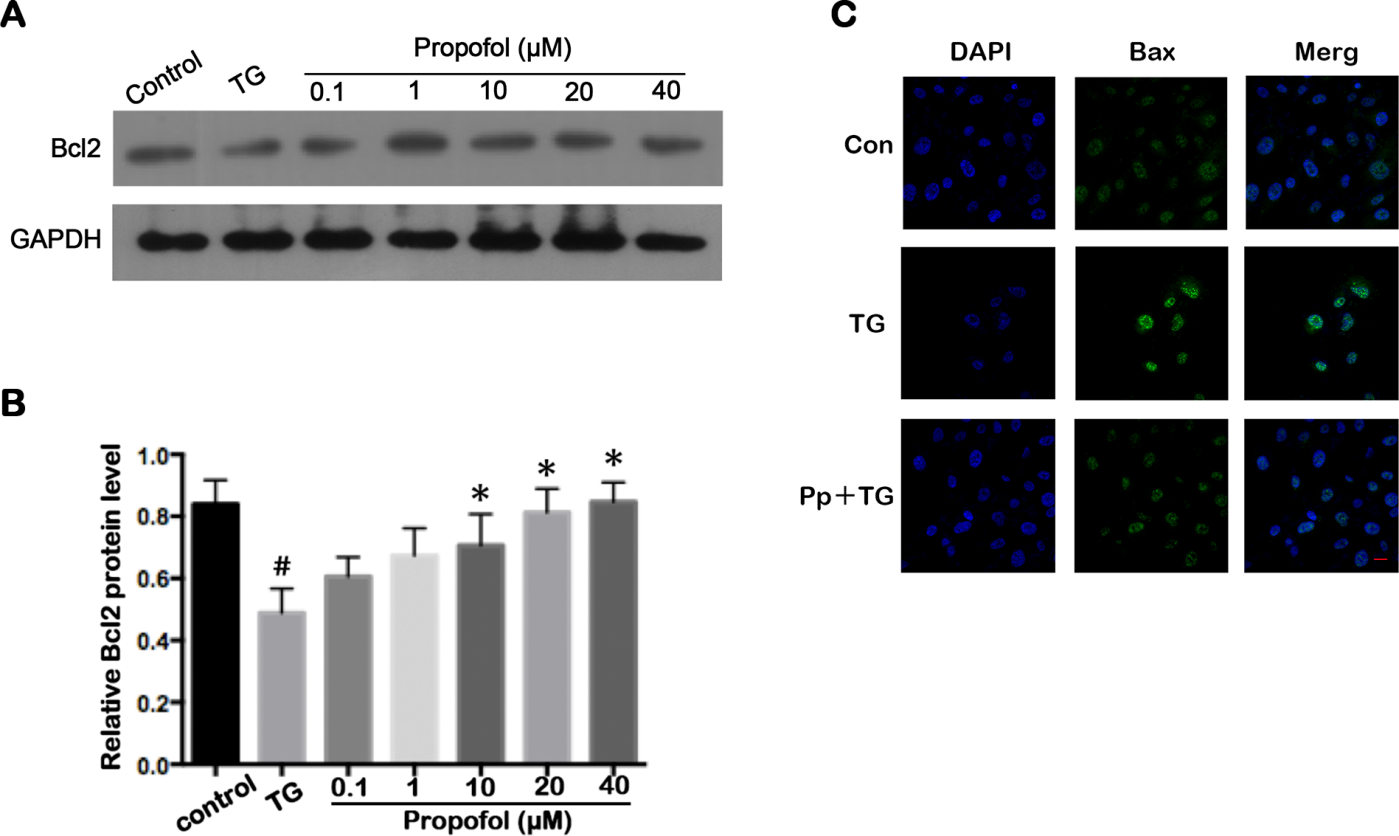
Propofol effect on apoptosis-associated proteins in TG-treated ARPE-19 cells. (A) Representative western blot for Bcl2. (B) Propofol attenuated the Bcl2 downregulation induced by TG in ARPE-19 cells. ARPE-19 cells were precubated with propofol for 12 h at different concentration, the treated with 1 μM TG for 12 h. The data are presented as the mean ± SEM from three independent experiments. Statistical analysis was performed by one-way analysis of variance with all pairwise multiple comparison procedures done by Tukey test. ^#^*p*<0.05 versus control group. ^*^p<0.05 versus TG group. (C) Immunofluorescence assay was used to locate the expression of Bax (green). Nuclei were marked with DAPI (blue). Scale bar = 20 μm

Bcl2 and Bax are central modulators of cell-intrinsic apoptosis [23]. Bcl2, an inhibitor of apoptosis, was noticeably downregulated by TG in ARPE-19 cells, whereas Bax was upregulated. These effects were reversed by pre-treatment with propofol for 24 h at a dose of 40 μM (Fig 4). These results indicated that propofol inhibited ARPE-19 cells apoptosis by modulating expression of Bcl2 (Fig 4A and 4B) and Bax (Fig 4C).

### Effect of propofol on BiP and CHOP expression in TG-treated ARPE-19 cells

**Fig 5:**
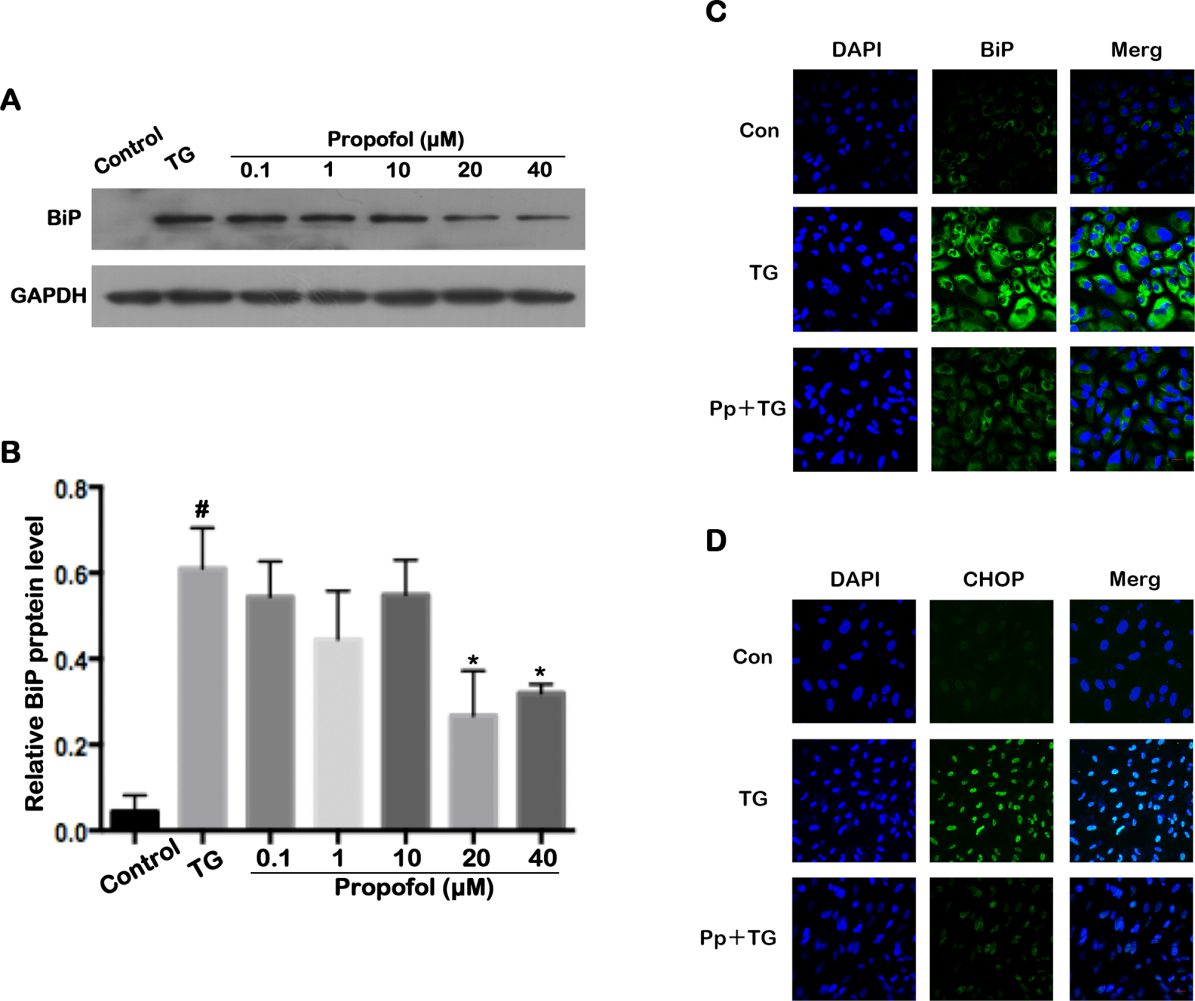
Propofol inhibits BiP and CHOP expression in TG-treated ARPE-19 cells. (A) Representative western blot for BiP. (B) Propofol significantly downregulated the BiP expression induced by TG at 20 and 40 μM. ARPE-19 cells were pretreated with different concentrations propofol for 12 h and treated with 1 μM TG for 12 h. The data are presented as the mean ± SEM from three independent experiments. Statistical analysis was performed by one-way analysis of variance with all pairwise multiple comparison procedures done by Tukey test. ^#^*p*<0.05 versus control group. ^*^p<0.05 versus TG group. (C-D) The expression of Bip and CHOP were evaluated by immunofluorescence assay in ARPE-19 cells after TG stimulation. Nuclei were labeled with DAPI (blue). Scale bar = 20 μm

Western blot analysis and confocal laser microscopy analysis were used to assess the effect of propofol on the expression of the ER stress-related proteins, BiP and CHOP. TG elevated expression of BiP and CHOP in ARPE-19 cells, demonstrating the effect of triggering ER stress (Fig 5). However, this elevated expression of Bip and CHOP was attenuated by pretreatment of the ARPE-19 cells with propofol at a concentration of 40 μM (Fig 5C and 5D). These results revealed that propofol modulated the expression level of Bip and CHOP.

### Effect of propofol on caspase 12 and caspase 3 expression in TG-induced ARPE-19 cells

**Fig 6:**
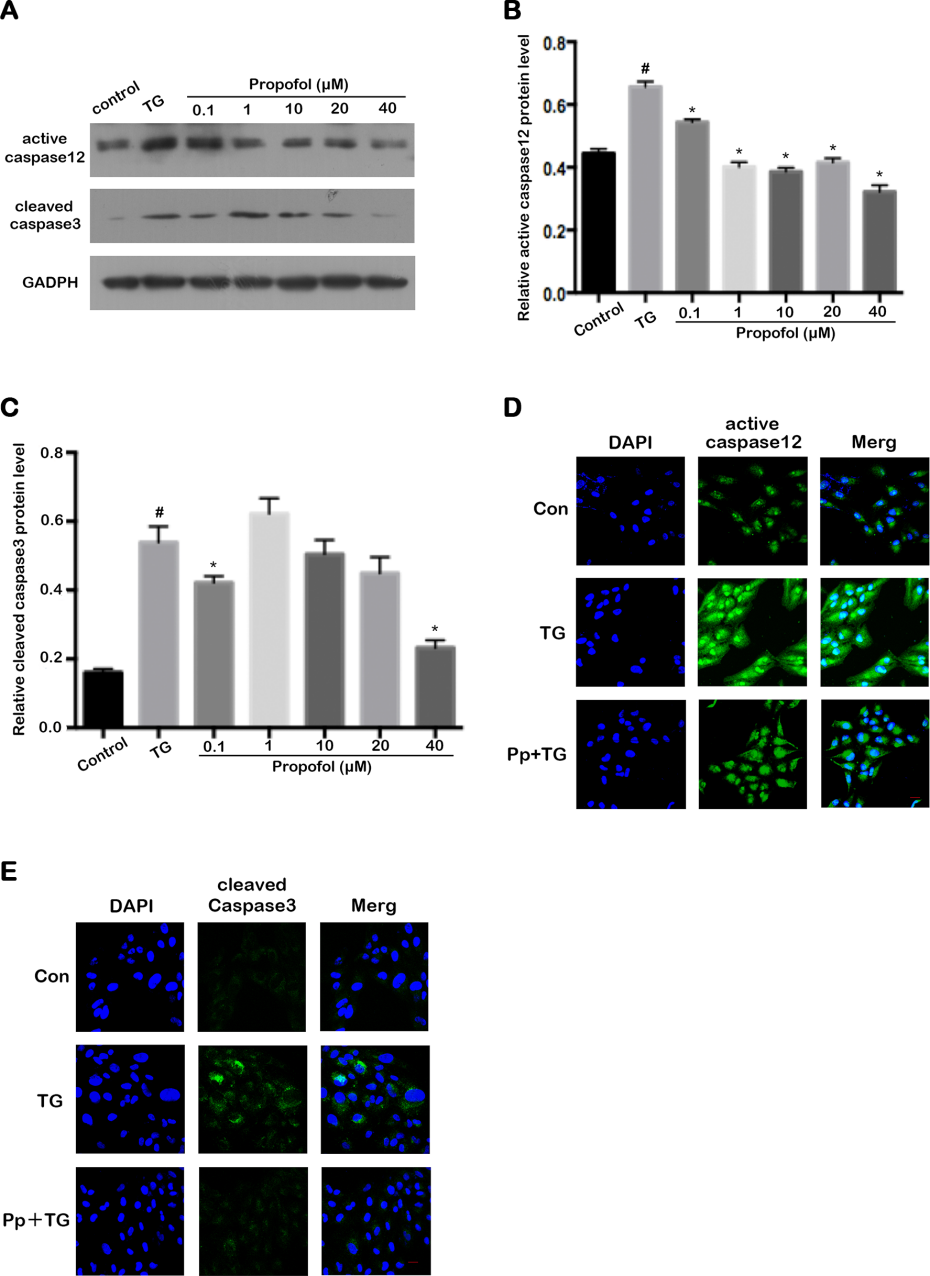
Propofol inhibits the acitivation of caspase 12 and caspase 3 in TG-induced ARPE-19 cells. (A) Representative western blot for active caspase 12 and cleaved caspase 3. (B-C) Propofol significantly downregulated the active caspase 12 level induced by TG in ARPE-19 cells. The level of cleaved caspase 3 in TG-treated ARPE-19 cells was downregulated by propofol at 0.1 and 40 μM. ARPE-19 cells were pretreated with different concentration propofol for 12 h and then subjected to 1μM for 12 h. The data are presented as the mean ± SEM from three independent experiments. Statistical analysis was performed by one-way analysis of variance with all pairwise multiple comparison procedures done by Tukey test. ^#^*p*<0.05 versus control group. ^*^p<0.05 versus TG group. (D-E) Representative photographs of active caspase 12 and cleaved caspase 3 in ARPE-19 cells from immunofluorescence assay are shown. ARPE-19 cells were pretreated with 40 μM propofol for 12 h and then exposed to 1μM TG for 12 h. Nuclei were labeled with DAPI (blue). Scale bar = 20 μm

Apoptosis mediated by ER stress is initiated by the activation of caspase 3 and caspase 12, where caspase 12 is the upstream factor of cleaved caspase 3 [24]. Western blot analysis and confocal laser microscopy analysis indicated that propofol pretreatment attenuated the TG-induced upregulation of these proteins ARPE-19 cells (Fig 6). The expression levels of activation of caspase 12 (Fig 6A, 6B and 6D) and caspase 3 (Fig 6A, 6C and 6E) were modulated by propofol.

### Effect of propofol on the ER stress relevant molecules in TG-treated ARPE-19 cells

**Fig 7:**
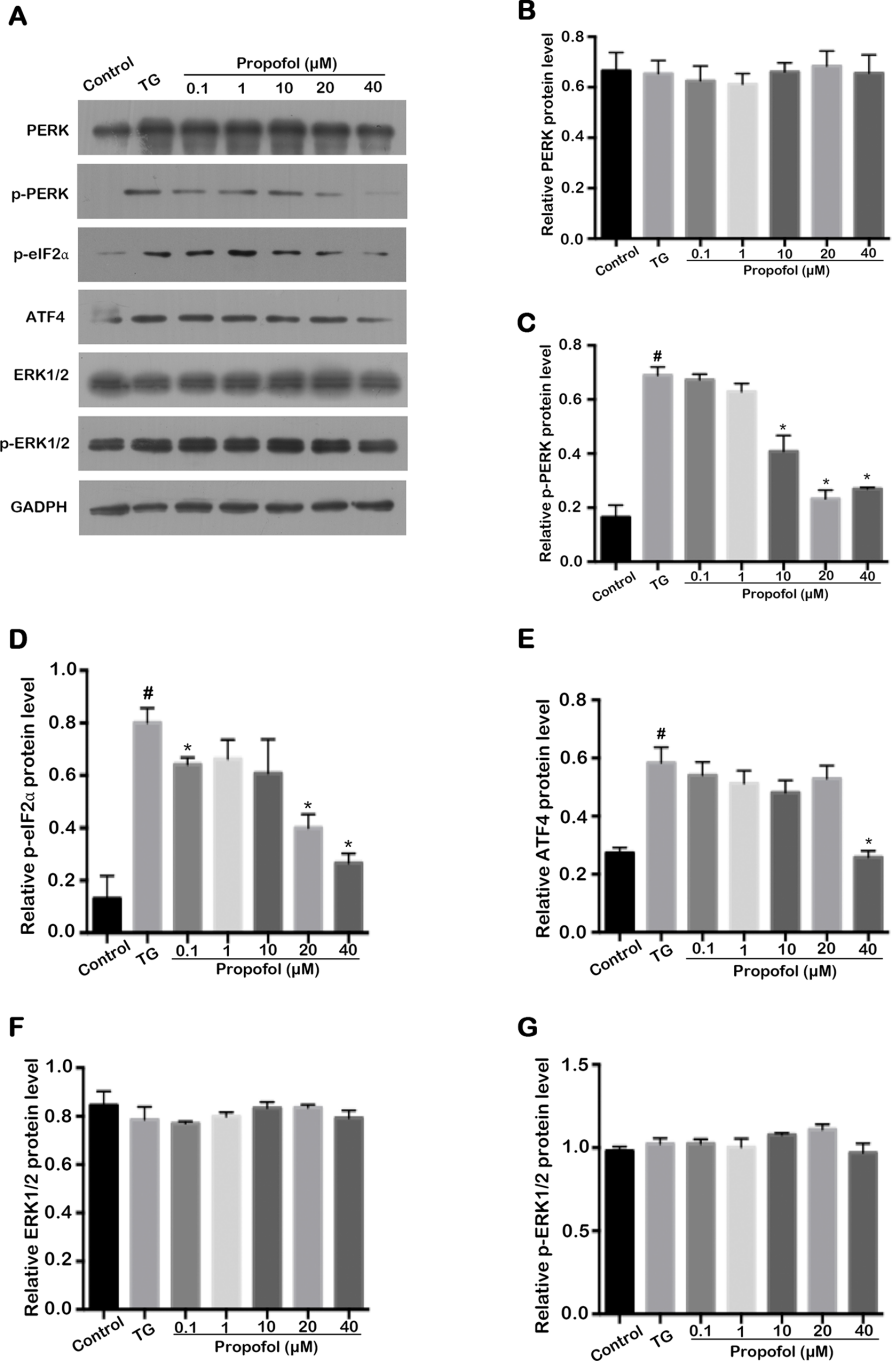
The ER stress relevant molecules were inhibited by propofol in TG-treated ARPE-19 cells. (A) Representative western blot for PERK, p-PERK, p-eIF2α, ATF4, ERK1/2 and p-ERK1/2. ARPE-19 cells were pretreated with different concentrations propofol for 12 h and then exposed for 12 h to 1μM TG. (B) Propofol had no effect on PERK level in TG-treated ARPE-19 cells. (C) Propofol decreased the p-PERK level in TG-treated ARPE-19 cells at the concentration of 10, 20, and 40 μM. (D) The p-eIF2α level induced by TG was downregulated by propofol at 0.1, 20, and 40 μM. (E) Propofol decreased the ATF4 expression in TG-stimulated ARPE-19 cells at 40 μM. (F-G) ERK1/2 and p-ERK1/2 level were not affected by propofol in TG-stimulated ARPE-19 cells. The data (B-G) are presented as the mean ± SEM from three independent experiments. Statistical analysis was performed by one-way analysis of variance with all pairwise multiple comparison procedures done by Tukey test. ^#^*p*<0.05 versus control group. ^*^p<0.05 versus TG group.

**Fig 8:**
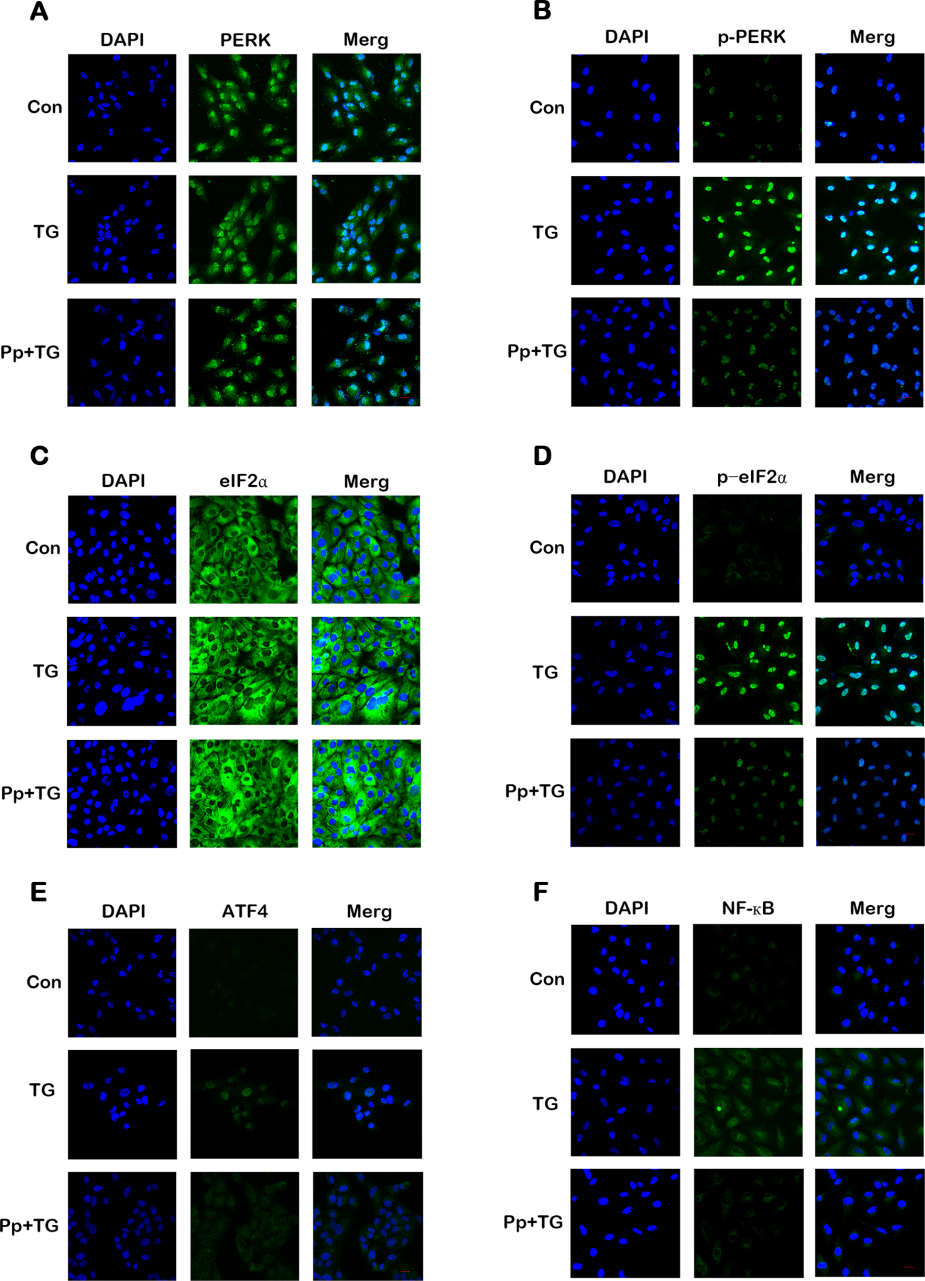
Propofol inhibits ER stress-associated proteins induced by TG with immunofluorescence assay. (A-F) A series of representative images of PERK, p-PERK, eIF2α, p-eIF2α, ATF4 and NF-κB from immunofluorescence assay in three groups are shown. ARPE-19 cells were pretreated with 40 μM propofol for 12 h and then exposed to 1μM TG for 12 h. Nuclei were labeled with DAPI (blue).

Apoptosis caused by ER stress is mediated partly by signaling through activation of PERK and eIF2α via phosphorylation. Western blot analysis and confocal laser microscopy analysis demonstrated that the TG-induced PERK (Fig 7A–7C and Fig 8A and 8B) and eIF2α phosphorylation (Fig 7A, 7D and Fig 8C and 8D) were attenuated by propofol pretreatment as was expression of another pro-death factor, ATF4 (Figs 7A, 7E and 8E). In addition, expression of TG-induced NF-κB decreased when cells were pretreated with propofol (Fig 8F). However, propofol pretreatment had no effect on the expression levels of ERK1/2 (Fig 7A and 7F) and p-ERK1/2 (Fig 7A and 7G). These results indicated that propofol protected ARPE-19 cells from TG-induced ER stress by modulating PERK- eIF2α- ATF4 signaling pathway and down-regulating the expression of NF-κB.

### Effects of propofol and TG on intracellular calcium in ARPE-19 cells

**Fig 9:**
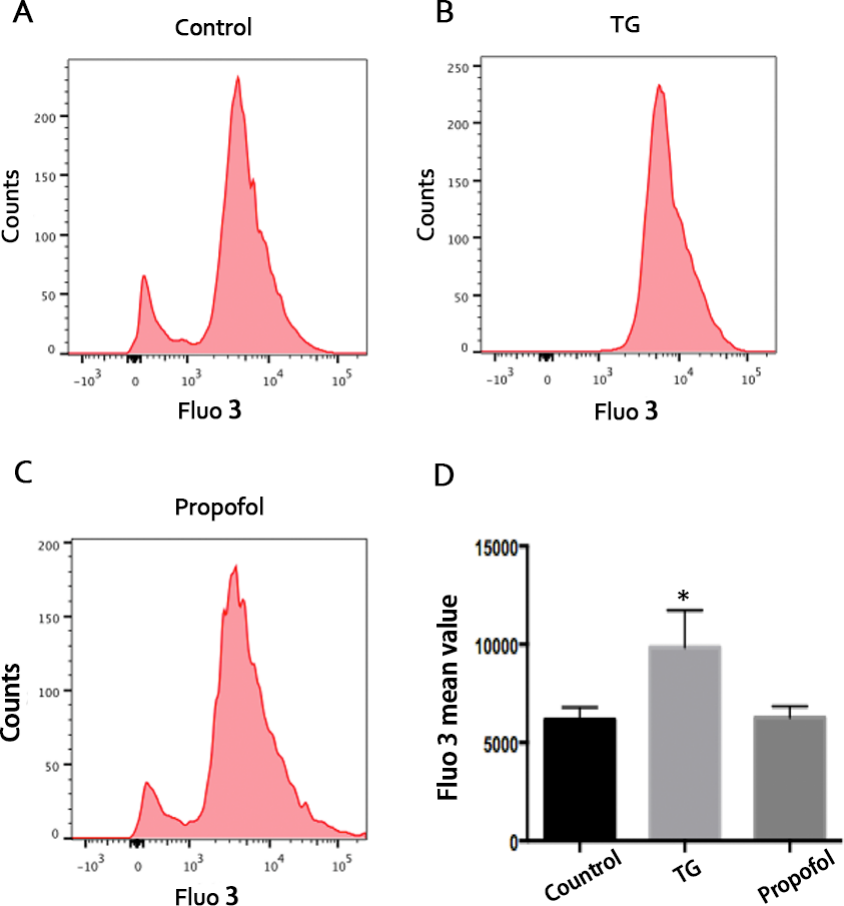
Analysis of intracellular calcium in ARPE-19 cells treated with TG and propofol. (A-C) ARPE-19 cells underwent TG and propofol for 12 h. The effects of TG and propofol on intracellular calcium were determined using flow cytometric analysis with the Fluo-3/AM. (D) The mean intensity of ARPE-19 cells treated with 1 μM TG and 40 μM propofol for 12 h. The experiment was repeated for three times, with each treatment in triplicate. Statistical analysis was performed by one-way analysis of variance with all pairwise multiple comparison procedures done by Tukey test. ^*^p<0.05 versus control group.

The intracellular calcium level increased during early and late stages of apoptosis[25]. It was demonstrated that apoptosis and biochemical changes induced by TG are likely a result of increase in intracellular calcium[26]. However, TG is a prototypical and powerful inducer of ER stress[27]. To investigate whether the protective effect of propofol on TG treated-ARPE 19 cells is associated with decreased intracellular calcium, we determined to use flow cytometric analysis of cells were stained with Fluo-3 AM. As shown in Fig 9B and 9D, TG caused an increased in the level of intracellular calcium within 12 h. However, the intracellular calcium level was not changed by propofol within 12 h (Fig 9C and 9D). Taken together, these results indicated that the protective effect of propofol on TG treated-ARPE 19 cells was not associated with change of intracellular calcium level.

## Discussion

ER stress is a major contributor to pathogenesis of AMD pathogenesis [6]. ER stress and the UPR, which can be initiated by AMD risk factors such as oxidative, proteotoxic, and metabolic stress, and the presence of cytokines, have been studied in the context of retinal degeneration, and particularly in regard to misfolded proteins in the RPE [28]. The major functions of UPR are maintaining regular ER function and protecting cells from environmental damage [29], and much of this and the ER stress-associated pathway is regulated by BiP [30, 31]. This important ER chaperone prevents accumulation of misfolded and unfolded proteins [32]and its expression is triggered by ER stress and misfolded proteins [33]. In our study, the expression level of BiP was elevated in TG-treated ARPE-19 cells. However, we also observed that propofol pretreatment attenuated BiP protein expression, and suggest that propofol can protect ARPE-19 cells from ER stress by reducing the accumulation of unfolded and misfolded proteins.

When ER stress is activated, PERK is oligomerized and phosphorylated by itself and eIF2α is phosphorylated by PERK. This phosphorylation inhibits reactivation of eIF2α resulting in a global arrest of protein synthesis that, in turn, prevents further influx of ER client proteins. In addition to reducing translation, PERK-mediated phosphorylation of eIF2α induces preferential translation of activating ATF4 [34–36]. CHOP is a major target object of the ATF4 transcription factor and the executor of cell apoptosis during ER stress [37].

In our study, we observed an increase of CHOP expression level in ARPE-19 cells after treatment with TG but that this effect was attenuated by propofol pretreatment. These data suggested that propofol suppressed the apoptosis activated by ER stress through downregulation of CHOP expression levels. TG-induced upregulation of p-PERK, p-eIF2α, and ATF4 was attenuated in the propofol pretreatment group. This suggested to us that the viability of TG-treated ARPE-19 cells was enhanced by propofol through suppression of the PERK-eIF2α-ATF4 signaling pathway.

The Bcl2 family regulate apoptosis [38]. Although Bcl2 family proteins are thought to localize in mitochondria, they also are found on the ER and nuclear membranes [39]. Antiapoptotic Bcl2 family members such as Bcl2, Mcl1, and BclxL along with the pro-apoptotic factors Bax, Bak, and Bik are localized at the ER [40]. Control of calcium homeostasis is one of the most documented functions of Bcl2 family members at the ER [41] and Bcl2 overexpression and Bax knockout in cells results in a decreased steady state ER calcium content [42]. Bcl2 family members adjust their sensitivity to diverse death stimuli through the regulation of ER calcium levels. However, Bcl2 family members can also affect ER calcium homeostasis by modulating UPR signaling. Bax and Bak form a protein complex with the cytosolic domain of IRE1α and modulate the amplitude of IRE1α signaling without affecting PERK activity [43]. Work by Hetz and colleagues demonstrated that deficiency in Bax and Bak clearly inhibited cell death, and also affected the ability of cells to mount a robust UPR with decreased splicing of XBP-1, as observed in ER stress-induced Bax/Bak^-/-^ double-knockout mice [43]. Several studies have shown that propofol protection against neuronal apoptosis is related to the increased expression of Bcl2 and decreased expression of Bax [44–46]. In our experimental cell model, TG-treatment reduced Bcl2 expression in ARPE-19 cells, and effect that was attenuated by propofol pretreatment. This pretreatment also attenuated Bax activity in cells treated by TG. Our data suggested that the apoptotic effects induced by ER stress were inhibited by propofol via the regulation of Bcl2 and Bax expression. The high expression of Bcl2 and the low expression of Bax that is induced by propofol inhibited cell apoptosis following ER stress, and possibly prolonged UPR signaling via the regulation of ER calcium levels.

The caspase family acts as central regulator in a number of apoptosis signaling pathways. Caspase 12 was found to be the initiator caspase in ER stress [47]. ER stress caused by TG and tunicamycin activate caspase 12, which then initiates the caspase-dependent apoptosis procedure [47]. Caspase 12-deficient cells are resistant to inducers of ER stress, suggesting that caspase 12 plays a major role in ER stress-induced apoptosis [48]. Caspase 12 initiates the ER-specific caspase cascade in a direct manner, and cleaves procaspase 9 at the processing site to activate caspase 9. Activated caspase 9 then activates caspase 3, the major effector caspase responsible for the destruction of various substrates [49]. In our study, we confirmed that TG not only significantly induced ARPE-19 cell apoptosis but also increased active caspase 12 and cleaved caspase 3 activities. When compared with TG-treated ARPE-19 cells, propofol-pretreated cells had a smaller apoptotic index, including a lower level of TUNEL-positive cells and PI-positive cells. Active caspase 12 and cleaved caspase 3 expression was also downregulated by propofol pretreatment. Propofol can significantly decrease cleaved caspase 3 activity and reduce apoptosis to a significant extent in neurodegenerative disease. Our data also suggest that propofol prevents the TG-induced activation of ARPE-19 cell apoptosis, and that the anti-apoptotic effect may be regulated by cellular signaling of the caspase 12-caspase 3 pathway. However, the expression of cleaved caspase 3 was upregulated by propofol pretreatment at 1 μM in Fig 6C. We speculated that cleaved caspase 3 is an apoptosis factor, which may be involved in different signal pathways, but play different roles in different signal pathways. Many reports have indicated that propofol augmented caspase-3 activation in relative concentration[50, 51]. The reason of upregulation of cleaved caspase 3 is that propofol may augment caspase 3 activation at 1 μM through different mechanism.

NF-κB is a major influence in modulating cellular responses when cells are subjected to stress or stimulation. There is accumulating evidence to suggest that ER stressors activate NF-κB in several cell lines [52]. Researchers have also reported that NF-κB is activated by phosphorylation of eIF2α induced by p-PERK [53]. In our study, we also observed that expression of NF-κB increased in TG-treated ARPE-19 cells and that significant attenuation was observed in the propofol-pretreated cells. A corresponding observation was made for phosphorylation of eIF2α by p-PERK. It is reasonable to conclude that our observation of NF-κB activation after treatment with TG was associated with the activation of the PERK/eIF2α pathway. Our results further suggest that propofol also attenuates NF-κB expression in TG-treated cells through suppression of the PERK/eIF2α signaling pathway.

MAPKs play an important role in regulating inflammation and apoptosis of cells. ERK1/2, a major member of the MAPKs, is implicated as an important modulator of cell apoptosis. Previous research has found that activation of ERK1/2 can attenuate ER stress [54]. However, in our study, we observed that expression levels of ERK1/2 and p-ERK1/2 were no different in the control and TG-treated groups. This suggests that TG-induced ER stress does not involve regulation of the ERK1/2 signaling pathway in ARPE-19 cells. Neither did propofol pretreatment affect levels of ERK1/2 and p-ERK1/2, implying that propofol does not influence apoptosis via this signaling pathway.

TG treating cells have already been widely used to study the mechanism of ER stress inducing apoptosis[55]. TG also has been used to stimulate RPE cells in order to research pathogenesis of AMD[56]. TG induces ER stress by inhibiting sarcoplasmic/endoplasmic reticulum calcium ATPase (SERCA), thus leading to severe depletion of ER calcium and increased intracellular calcium[26]. In our research, TG also increased intracellular calcium in ARPE-19 cells within 12 h. However, the intracellular calcium was not obviously change in propofol-treated ARPE-19 cells within 12 h. These results indicated that the protective effect of propofol on TG-treated ARPE-19 cells was not by changing intracellular calcium level.

In conclusion, ARPE-19 cells treated with TG underwent apoptosis through the activation of ER stress. Pretreatment with propofol caused a significant decrease in BiP expression and reduced NF-κB activation. Additionally, our findings revealed that propofol inhibited PERK-eIF2α-ATF4-CHOP activation under ER stress. It may also prevent TG-induced apoptosis of ARPE-19 cells via the suppression of the caspase 12-caspase 3 pathway. Therefore, we conclude that propofol does not affect ER stress-induced apoptosis via a single mechanism.

Undoubtedly, there are some limitations of our study; for example, some other ER stress inducers, such as tunicamycin, should be evaluated in future studies. We will do further study to investigate whether the physiological antagonism of TG has some effect on RPE cells apoptosis. Future studies should also explore the potential efficacy of propofol against ER stress-induced retinal degeneration via the apoptosis of ARPE-19 cells. Nevertheless, our study offers a deeper understanding of propofol’s potential for clinical application, in addition to the protective mechanism of propofol.
